# Inpatient Growth in Infants Requiring Pharmacologic Treatment for Neonatal Opioid Withdrawal Syndrome

**DOI:** 10.1155/2024/2212688

**Published:** 2024-08-24

**Authors:** Ashajyothi M. Siddappa, Erin Morris, Michael D. Evans, Sarah Pelinka, Constance Adkisson

**Affiliations:** ^1^ Neonatology Department of Pediatrics Hennepin Healthcare, Minneapolis, Minnesota, USA; ^2^ Department of Pediatrics University of Minnesota, Minneapolis, Minnesota, USA; ^3^ Division of Neonatology University of Minnesota, Minneapolis, Minnesota, USA; ^4^ Clinical and Translational Science Institute University of Minnesota, Minneapolis, Minnesota, USA

**Keywords:** growth trajectories, head circumference, length, neonatal opioid withdrawal syndrome, opioids, weight, Z-score

## Abstract

**Aim:** To assess inpatient growth parameter trajectories and to identify the type of opioid exposure and treatment characteristics influencing growth parameters of infants admitted to the newborn intensive care unit (NICU) for pharmacological treatment of neonatal opioid withdrawal syndrome (NOWS).

**Methods:** Charts of term infants with NOWS admitted to NICU from 2012 to 2019, who received pharmacologic treatment, were reviewed. Intake (volume: mL/kg/day; calorie: kcal/kg/day) and growth parameter trajectories (weight, head circumference, and length) were analyzed based on the type of prenatal opioid exposure (short-acting opioids (SAOs), long-acting opioids (LAOs), and polysubstance), pharmacologic treatment, and sex. Growth measurement patterns over time were compared between groups using longitudinal mixed-effects models.

**Results:** One hundred nineteen infants were included in the study with median birth weight *Z*-score of −0.19 at birth and decreased to a median of −0.72 at discharge. Exposure to SAO was associated with an increase in *Z*-scores nearing discharge across all growth parameters (*Z*-score for weight *p* = 0.03). Polysubstance exposure was associated with a decrease in *Z*-scores for length and head circumference throughout hospitalization. Infants with adjunct clonidine treatment had an increase in *Z*-score for weight trends. Male infants had a decrease in *Z*-scores for weight (male −0.96, female −0.59, interaction *p* = 0.06) and length (male −1.17, female −0.57, interaction *p* = 0.003) at Day 28. Despite the difference in growth trajectories, intake in terms of amount (mL/kg/day) and calorie intake (kcal/kg/day) was similar based on prenatal exposure, treatment, and sex.

**Conclusion**: Infants with NOWS requiring pharmacologic treatment have a decrease in *Z*-scores for weight, length, and head circumference at birth and at hospital discharge. Infants with prenatal polysubstance exposure were at particular risk for poorer inpatient growth relative to infants exposed to SAO and LAO, indicated by lower *Z*-scores for length and occipital frontal circumference (OFC).

## 1. Introduction

Prenatal opioid exposure has reached an epidemic proportion in the last decade [1]. This has led to an increase in the number of infants admitted to the newborn intensive care unit (NICU) for treatment of neonatal opioid withdrawal syndrome (NOWS) [2–4]. The incidence of NOWS ranges from 50% to 80% in infants exposed to opioids, and infants usually develop withdrawal symptoms within 2–5 days after birth [1, 3, 5, 6]. NOWS is characterized by a spectrum of withdrawal symptoms involving the central nervous system, autonomic nervous system, and gastrointestinal (GI) tract [5, 6]. Symptoms related to increased excitability of the central nervous system include hypertonia, excessive crying, tremors, sleep disturbances, and seizures; autonomic symptoms include sweating, sneezing, and respiratory distress; and GI symptoms include poor feeding, vomiting, and watery stools [7]. Studies have shown that exposure to methadone causes severe symptoms of NOWS when compared with heroin exposure [8]. Similarly, infants exposed to polysubstance have severe NOWS symptoms [9]. Typically, infants who exhibit severe withdrawal symptoms not responsive to nonpharmacologic measures alone are transferred to the NICU for pharmacologic treatment.

Exposure to opioids during pregnancy alters placental blood flow affecting nutrient delivery to fetus leading to fetal growth restriction and low birth weight [7, 10–12]. Pregnant women with opioid use disorder are at risk for poor nutrition, use of other illicit substances including smoking, and decreased access to and consistency of prenatal care; all these factors contribute to poor fetal growth [12, 13]. Opioid use during pregnancy can alter maternal physiology and lead to conditions like hypertension, seizures, pre-eclampsia, preterm labor, and fetal growth restriction [14]. Common opioids used during pregnancy include illicit drugs such as heroin, as well as prescription pain medications such as oxycodone, hydrocodone, codeine, morphine, methadone, and buprenorphine. Buprenorphine and methadone are the two long-acting opioids (LAO) used as the standard of care to treat pregnant women who are addicted to opioids before, during, and after pregnancy (ACOG) [1, 7, 12, 15].

Infants with NOWS admitted to NICU are at risk for growth failure and have been described as having excessive postnatal weight loss [16]. Feeding remains a challenge, and feeding difficulties are common in infants exposed to prenatal opioids (18.1%) when compared with non–opioid-exposed infants (3%) [17]. NOWS infants exhibit many behaviors which include nipple rejection, hiccoughing, spitting up, coughing, averting their face, turning away, grimacing, hyperextension of arms or legs, flailing of arms, pushing or spitting out the nipple, and vocal objections like whimpering; all these behaviors lead to disorganized and ineffective sucking in turn to poor oral intake [18, 19]. Infants with NOWS presenting with severe GI symptoms including diarrhea and vomiting or regurgitation are at risk for poor postnatal weight gain [16]. The symptomatic NOWS infants with poor feeding and poor oral intake fail to meet increased metabolic demands leading to poor weight gain [16]. Several growth patterns have been documented in infants with NOWS, ranging from weight loss and failure to thrive to increased weight gain [16, 19, 20]. Studies on growth during the inpatient period in infants undergoing treatment for NOWS are limited. The aim of this study is to examine growth parameter trajectories of infants with NOWS admitted to the NICU for pharmacologic therapy and to identify the type of opioid exposure and treatment characteristics that may put infants with NOWS at risk for poor inpatient growth.

## 2. Materials and Methods

### 2.1. Study Population

This is a retrospective review approved by the Institutional Review Board (IRB) of the hospital. The electronic medical records of infants with NOWS diagnosis delivered between January 2012 and January 2019 were reviewed. Only infants born at term gestation (≥ 37-week gestation) admitted to NICU for pharmacological treatment were included in the study.

### 2.2. Measurements

Data collected included maternal age; maternal comorbidities; type of delivery; sex; type of drug exposure obtained from the maternal report or from the results of meconium toxicology screen; gestational age at birth and at discharge; and anthropometric parameters including daily weights, weekly head circumference (occipital frontal circumference [OFC]), and length measurements from NICU admission to discharge, length of stay (LOS), medications used for treating NOWS, and duration of therapy. Feeding data collected included type of feeding (oral, gavage, or IV fluids), type of milk used for feeding (breast milk, formula), twice weekly interval collection of intake amount (mL/kg/day), and caloric intake (kcal/kg/day).

Infants were monitored for withdrawal symptoms following admission to NICU using neonatal withdrawal inventory (NWI). Before starting medication, nonpharmacological measures were optimized (swaddling/skin-to-skin care, decreased noise, light, stimulation, and feeding maternal breast milk as available). During the study period, our NICU could not accommodate rooming in with mothers for these infants. Breast milk was offered to infants born to mothers who were free of illicit drug use for 30 days prior to delivery, if mothers were enrolled in a methadone program and were HIV negative. Infants were started on pharmacological treatment if they had three NWI scores of ≥ 8 or two scores of ≥ 12. Infants were started on medications per unit protocol. All infants needing pharmacological treatment were started on oral morphine solution first and dose escalation, and adjunct therapy with clonidine and/or phenobarbital was done based on symptom control based on NAS scores. Ten to twenty percent of a total dose of morphine received per day was weaned once symptoms were controlled based on lower NAS scores. Prior to discharge, infants were weaned off scheduled morphine first, and then, clonidine was weaned. Morphine was used as needed while clonidine was weaned. Infants were eligible for discharge 48 h after the last dose of morphine.

### 2.3. Statistical Methods

Demographic and clinical characteristics were summarized using counts and rates or means and standard deviations (SDs). As a control group for NOWS was not available, infants were compared to WHO *Z*-score norms for age and sex. Weight, length, and head circumference measurements were converted to weight-, length-, and OFC-for-age *Z*-scores using the WHO Child Growth Standards [21]. Type of opioid exposure during pregnancy was classified as short-acting opioid (SAO) exposure (oxycodone, fentanyl, hydrocodone, and heroin), LAO exposure (buprenorphine and methadone), and polysubstance exposure (exposure to two or more of cocaine, methamphetamine, THC, benzodiazepine, gabapentin, nicotine, and/or alcohol). This was done to identify the type of opioid exposure and treatment characteristic influencing growth parameters in infants with NOWS.

Growth trajectories were compared between groups: drug exposure (SAO vs LAO vs SAO+LAO vs polysubstance), treatment (morphine vs morphine + clonidine), sex (male vs female), and breast milk use (yes or no), using longitudinal mixed-effects models with natural cubic splines for age, with fixed effects terms for age, group, age-by-group interaction, and gestational age at birth, and random effects terms for intercept and age by infant. Intake measurements (volume: mL/kg/day and caloric: kcal/kg/day) were compared by exposure and treatment groups using mixed-effects models with fixed effects for group, time and group-by-time interaction, and random effects term for infant. Analyses were conducted using R version 4.1.1 (R Foundation for Statistical Computing, Vienna, Austria).

## 3. Results

There were 133 infants admitted to the NICU with a diagnosis of NOWS for treatment during the study period. Fourteen infants were excluded as their gestational age was less than 37 weeks at birth. For the 119 infants meeting the inclusion criteria, maternal and infant characteristics are shown in Table 1. Forty-two percent (*n* = 50) of infants were born to mothers with incomplete prenatal care, which included limited prenatal care (≤ 4 prenatal visits), late prenatal care (after 20 weeks), and no prenatal care. The mean length of NICU stay was 28 days.

The average weight gain per day during hospital course was 20 g (SD 15.8), and *Z*-score per day mean was −0.027 (SD 0.030). For all opioid-exposed infants, there was a trend in decreasing *Z*-score for weight during the hospital stay. There was a similar trend in decreased OFC and length (Table 1).

The type of opioid exposure and pharmacologic therapies and duration of therapy for our population are displayed in Table 2. The mean LOS (days) based on exposure (overall *p* value = 0.09) for SAO (*n* = 8) was 18 (SD10), for LAO (*n* = 39) was 29 (SD 14), for SAO and LAO (*n* = 17) was 33 (SD 18), and for polysubstance (*n* = 55) was 31 (SD 16).

**Figure 1:**
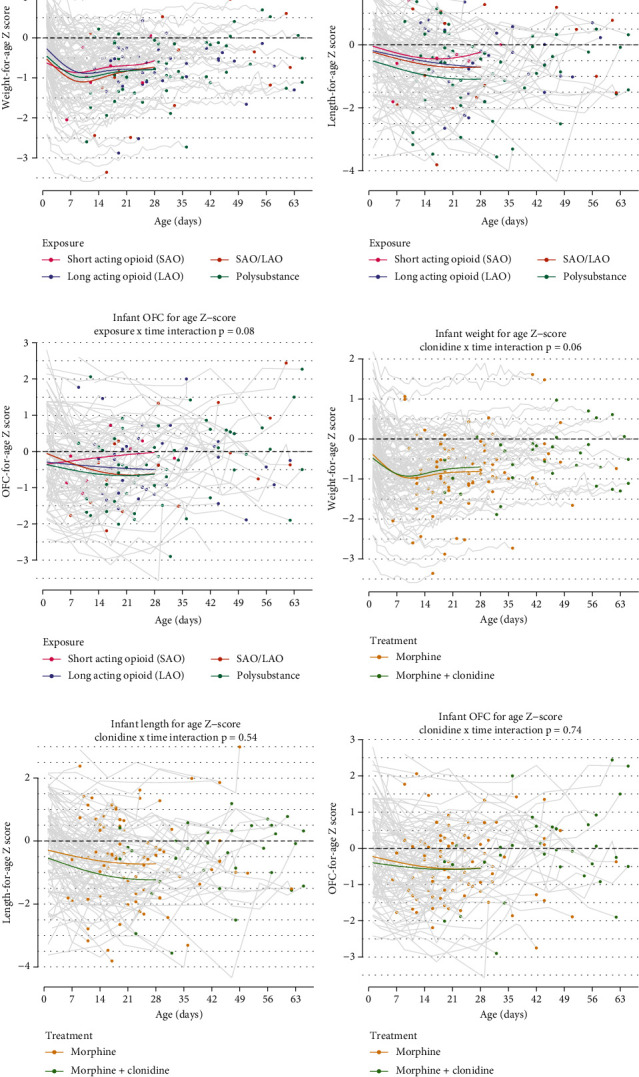
NOWS infant growth trajectories. (a) SAO associated with improved weight *Z*-score throughout hospitalization. (b, c) Polysubstance exposure is associated with the lowest *Z*-scores for length and head circumference (OFC) throughout hospitalization. (d) Adjunctive clonidine treatment had improved weight *Z*-score trends. (e, f) Clonidine had no significant impact on length or head circumference (OFC) throughout hospitalization.

Growth trajectories based on exposure and on pharmacologic treatment are displayed in Figure 1. Exposure to SAO was associated with the highest *Z*-scores nearing discharge across all growth parameters (Figures 1(a), 1(b), and 1(c); weight, length, and OFC), and there was statistical significance in this trend for weight (*p* = 0.03). Polysubstance exposure was associated with the lowest *Z*-scores for length and OFC throughout hospitalization (Figures 1(b) and 1(c)). Growth parameters analyzed based on treatment show downtrending length *Z*-scores across all treatment groups (Figure 1(e)). Infants treated with adjunctive clonidine had improved *Z*-score for weight over hospitalization, with a *p* value approaching significance at *p* = 0.06 (Figure 1(d)). Clonidine had no significant impact on length or head circumference (OFC) throughout hospitalization (Figures 1(e) and 1(f)).

**Figure 2:**
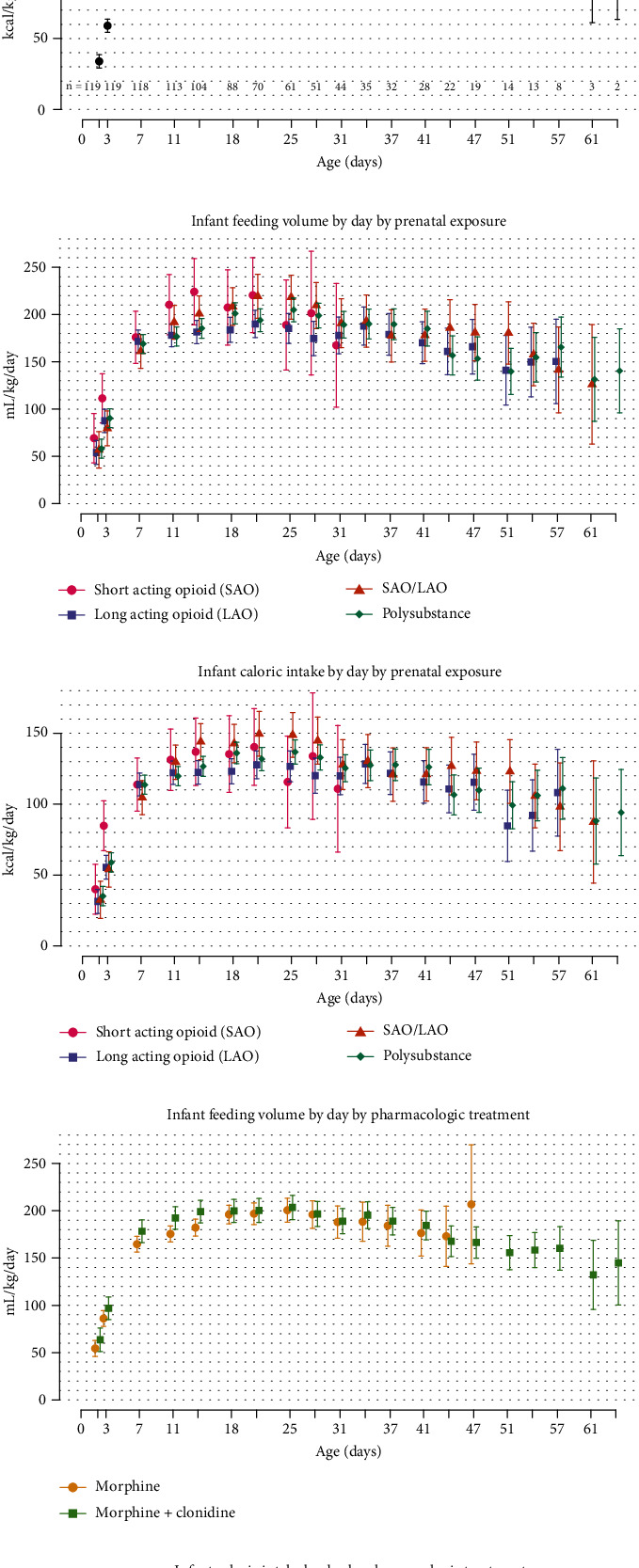
NOWS infant feeding volume and caloric intake. (a, b) Overall feeding volumes and caloric intake by day. (c, d) Feeding volumes and caloric intake were not different based on prenatal exposures. (e, f) Feeding volumes and caloric intake were not different based on pharmacologic treatment.

Figure 2 shows total fluid intake and caloric intake at twice weekly intervals (Figures 2(a) and 2(b)), which were not significantly different between sex, exposure (Figures 2(c) and 2(d)), or treatment group (Figures 2(e) and 2(f)). The growth trajectory differences were observed despite similar feeding volumes (mL/kg/day) and caloric intake (kcal/kg/day) across groups (Figure 1). The measured total fluid intake (mL/kg/day) and caloric intake (kcal/kg/day) at twice weekly intervals showed increased intake from day of life (DOL) 11 to DOL 41 during the hospital stay (Figure 2); there was no statistically significant difference in intake in terms of volume (mL/kg/day) and caloric intake (kcal/kg/day) between male and female infants (Table S1).

**Figure 3:**
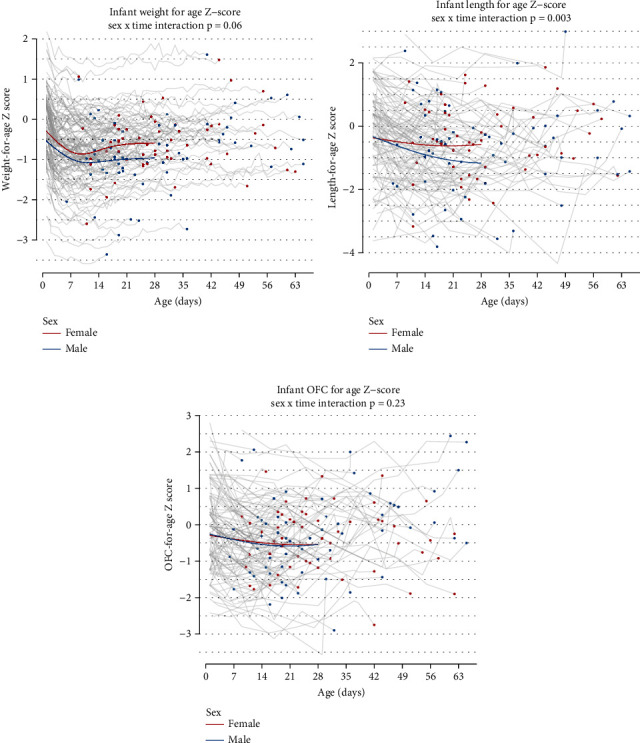
NOWS infant growth trajectories by sex. Graph shows male infants with lower *Z*-scores for (a) weight and (b) length. Both male and female infants showed similar trend in *Z*-score for (c) head circumference (OFC).

**Figure 4:**
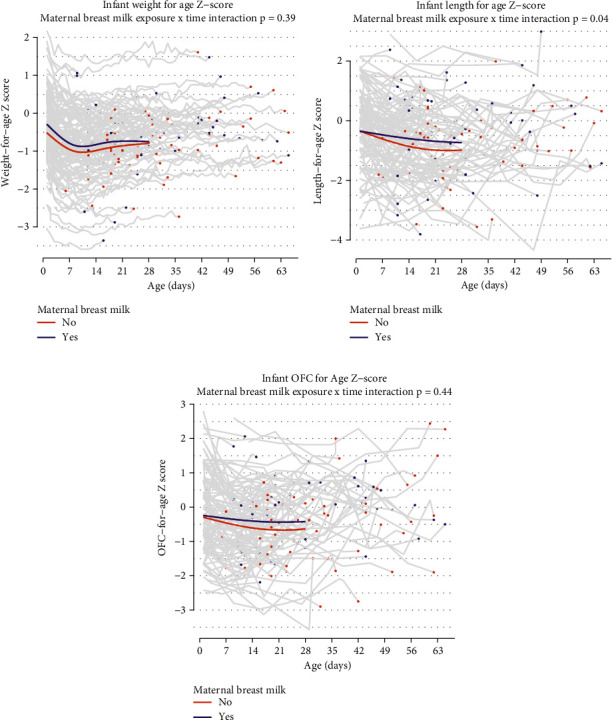
NOWS infant growth trajectories by any maternal breast milk provided. Graphs (a–c) show improved growth trajectories for infants with any breast milk exposure, with length being statistically significant (*p* = 0.04).

As shown in Figure 3, male infants had lower *Z*-scores for weight (male −0.96, female −0.59, interaction *p* = 0.06) and length (male −1.17, female −0.57, interaction *p* = 0.003) at 28 days. Breastfeeding and use of breast milk during the inpatient period in our study was 45% (*n* = 54) and at discharge was 42% (*n* = 50). Along with breast milk use and breastfeeding, these infants received supplementation with formula during their inpatient stay and at discharge. Regarding feeding practices in our study population, 20% (*n* = 24) of infants received fortification and 26% (*n* = 31) required gavage feedings during the first 2 weeks after admission. A positive *Z*-score trend for all growth parameters was seen for infants who received any maternal breast milk (*p* value of 0.04) (Figure 4).

## 4. Discussion

Our hospital experience supports the growing concern that infants with NOWS are at risk for inpatient growth failure, with increasingly negative *Z*-scores for weight, length, and OFC from birth to hospital discharge. We identified infants with prenatal polysubstance exposure to be at particular risk for poor inpatient growth despite commensurate intakes with other exposure histories. Polysubstance exposure has previously been identified as a risk factor for more severe withdrawal symptoms [9]. Additionally, we identified that adjunct treatment with clonidine may have a positive effect on inpatient weight trends, although the longer length of stay in these infants may be a confounder and allow an opportunity for inpatient catchup growth.

Bada et al. showed that prenatal exposure to opiates had a significant effect on birth weight (*p* < 0.05) and some effect on length (*p* < 0.08) independent of use of other drug use, prenatal care, or other medical risk factors [10]. Corr et al. did not show significant growth differences between birth and 1 year in infants with NOWS compared with control [22]. Our study complements a recent study by Favara et al., who also studied growth in infants with NOWS (*n* = 864) and found that infants were smaller at birth with median percentiles for weight at 30%, head circumference at 23%, and length at 37% and all growth parameters were significantly decreased at discharge with median percentiles at 12% for weight, 6.5% for head circumference, and 13% for length [23]. Additionally, Liu et al. studied the growth parameters of infants exposed to nicotine and/or opioids in pregnancy [24]. Liu et al.'s study showed significantly lower birth weight, length, and head circumference in infants born to opioid-dependent mothers than those infants born to smoking, non–opioid-dependent mothers, indicating opioid exposure impacts in utero growth. Our study adds to the analysis of these parameters by exposure and treatment group and highlights groups of infants with NOWS at higher risk for poor inpatient growth.

Previous studies evaluating feeding practices showed no significant difference between infants with NOWS receiving fortified versus standard formula regarding weight loss, days to regain birth weight, need for pharmacologic treatment, or length of hospital stay [ 8, 25]. However, in one study, infants on higher calorie formula had higher mean weight gain per day over the first 21 DOL [25]. Other studies have suggested that adequate nutrition to minimize weight loss should be part of the initial therapy for NOWS infants [8]. For some infants with NOWS who have inadequate weight gain, an increase in frequency of feedings with a high-calorie formula, lactose-free formula, or both may be required to mitigate some of the symptoms of NOWS [2, 5, 8]. In our study, 20% (*n* = 24) of infants received fortification, and 26% (*n* = 31) required gavage feedings.

Despite the growth failure noted in NOWS infants, hyperphagia can be observed in this population. While the cause of the hyperphagia is not known, it could be secondary to increased corticotrophin and due to increased metabolic demand during the withdrawal period and could indicate self-soothing behavior through feeding. Infants with NOWS, 26%–56%, present with hyperphagia during the second week of life and have severe withdrawal symptoms requiring pharmacotherapy [20, 26]. In our study, we noticed that NOWS infants had increased intake in terms of volume (> 170 mL/kg/day) and calorie (> 120 kcal/kg/day) from DOL 11 to DOL 41, and it included dietary intake as infants progressed to ad lib demand feedings, which may increase variability in our data. In our study, no difference was observed in terms of volume (mL/kg/day) and calorie (kcal/kg/day) between male and female infants. We noticed a decrease in *Z*-score trends for weight and length in male infants when compared with female infants despite similar feeding intake. Despite increased feeding volume and caloric intake in infants with NOWS in our study, there was a decrease in trend for growth parameter trajectories. A retrospective study by Shephard et al. showed that infants with NOWS (*n* = 48) needing treatment had hyperphagia (> 200 mL/kg/d) and excessive weight gain (20 g/day) [20]. Another study by Martinez, Kastner, and Taeusch noticed (*n* = 44) hyperphagia after birth by 2 weeks of life, and hyperphagia was not associated with increased weight gain, infant withdrawal score, or maternal methadone dose [26].

Studies have shown that male infants have severe NOWS symptoms when compared with female infants, which could explain the difference in growth trajectories seen in our study [1, 27]. The study by Unger et al. in a prospective randomized trial showed that male infants (*n* = 60 out of 131) in the study had higher birth weight (*p* = 0.027) and head circumference (*p* = 0.017) compared with female infants, but no sex difference was seen with regard to severity or with duration of symptoms [28]. Studies have shown breastfeeding, rooming in, and pharmacologic and nonpharmacologic interventions to help with optimizing weight gain and reducing the LOS in infants with NOWS [5, 14, 29–33]. Our study confirms the positive impact of breast milk use on growth trajectories including weight, OFC, and length. Our data surrounding breastfeeding also compliments existing evidence showing a decrease in severity and duration of NOWS symptoms when infants with prenatal exposure to methadone or buprenorphine are breastfed [7].

Studies evaluating early outpatient growth of infants with NOWS are limited. Infants with NOWS with prenatal and postnatal growth failure on top of prenatal exposure to opioids place them at elevated risk for neurodevelopmental impairment [23, 34, 35]. Long-term follow-up studies in infants with NOWS remains an ongoing challenge as these children's families are mobile and experience relapse in treatment and psychosocial stressors [35].

There are several limitations to our study; it is a single-center retrospective study with a small sample size. The results of our study may not be generalizable due to variability in the duration of in utero opioid exposure, type of opioid exposure, associated maternal comorbidities, and postnatal use of different treatment regimen and feeding regimen in infants with NOWS. There is also significant variability in the management of infants with NOWS across hospitals, and we acknowledge that our population could have unique growth trajectories based on our use of the NWI scoring tool and means of pharmacologic treatment. Despite these limitations, we find this data valuable in recognizing the risk that infants with NOWS may have toward poor inpatient growth.

## 5. Conclusion

Infants with NOWS requiring pharmacologic treatment have negative *Z*-scores by weight, length, and head circumference at birth and at hospital discharge as compared to WHO *Z*-score norms. Polysubstance exposure places these infants at particular risk for poor inpatient growth, and clonidine may improve inpatient growth trends. With a wide variation in the management of infants with NOWS, knowledge of specific risk factors for poor inpatient growth can aid providers in the optimization of feedings. We advocate for close monitoring of growth parameters for infants with NOWS as early growth failure could place them at additional neurodevelopmental risk.

## Figures and Tables

**Table 1:** Maternal and neonatal characteristics.

Male, *M* (%)	63 (53.0)
GA at birth, weeks (SD)	39.3 (1.3)
Race, *n* (%)	
African American	22 (19.0)
Native American	61 (51.3)
Caucasian	9 (8.0)
Hispanic	10 (8.4)
Unknown/other	17 (14.3)
APGAR 5 Min (SD)	8.6 (1.1)
Mode of delivery, C-section, *n* (%)	37 (31)
Mother's age, years (SD)	28.4 (5.1)
GA at discharge, weeks (SD)	43.5 (2.7)
DOL NICU admit, days (SD)	2.4 (1.7)
Length of hospital stay, days (SD)	30.0 (15.2)
Birth weight, kg (SD), *Z*-score (SD)	3.2 (0.43), −0.2 (0.9)
Discharge weight, kg (SD), *Z*-score (SD)	3.9 (0.79), −0.7 (0.8)
Birth OFC, cm (SD), *Z*-score (SD)	34.2 (1.5), −0.1 (1.2)
Discharge OFC, cm (SD), *Z*-score (SD)	36.3 (1.8), −0.4 (1.0)
Birth length, cm (SD), *Z*-score (SD)	49.3 (2.6), −0.2 (1.3)
Discharge length, cm (SD), *Z*-score (SD)	53.0 (3.2), −0.6 (1.3)

Abbreviations: DOL = day of life, GA = gestational age, OFC = occipital frontal circumference.

**Table 2:** Type of opioid exposure and treatment.

Exposure, *n* (%)	
SAO	8 (6.8)
LAO	39 (33.0)
SAO + LAO	17 (14.4)
Polysubstance	55 (47.0)
Treatment, *n* (%)	
Morphine start, DOL (SD)	3.2 (1.7)
Morphine duration of therapy, DOL (SD)	24.0 (15.2)
Morphine	71 (60.0)
Morphine + clonidine	20 (17.0)
Morphine + phenobarbital	15 (13.0)
Morphine + clonidine + phenobarbital	13 (12.0)

Abbreviations: DOL = day of life, LAO = long-acting opioid (buprenorphine and methadone), SAO = short-acting opioid (oxycodone, fentanyl, hydrocodone, and heroin).

## Data Availability

The data used to support the findings of this study are included within the article.

## References

[1] Conradt E., Crowell S. E., Lester B. M. (2018). Early life stress and environmental influences on the neurodevelopment of children with prenatal opioid exposure. *Neurobiology of Stress.*.

[2] Kraft W. K., Stover M. W., Davis J. M. (2016). Neonatal abstinence syndrome: pharmacologic strategies for the mother and infant. *Seminars in Perinatology.*.

[3] Patrick S. W., Schumacher R. E., Benneyworth B. D., Krans E. E., McAllister J. M., Davis M. M. (2012). Neonatal abstinence syndrome and associated health care expenditures: United States, 2000-2009. *Journal of the American Medical Association*.

[4] Gomez-Pomar E., Finnegan L. P. (2018). The epidemic of neonatal abstinence syndrome, historical references of its’ origins, assessment, and management. *Frontiers in Pediatrics*.

[5] Wachman E. M., Schiff D. M., Silverstein M. (2018). Neonatal abstinence syndrome. *JAMA*.

[6] Finnegan L. (2013). *Licit and illicit drug use during pregnancy: Maternal, neonatal and early childhood consequences*.

[7] Logan B. A., Brown M. S., Hayes M. J. (2013). Neonatal abstinence syndrome: treatment and pediatric outcomes. *Clinical Obstetrics and Gynecology*.

[8] Hudak M. L., Tan R. C., THE COMMITTEE ON DRUGS (2012). Neonatal drug withdrawal. *Pediatrics*.

[9] Sanlorenzo L., Stark A., Patrick S. W. (2018). Neonatal abstinence syndrome: an update. *Current Opinion in Pediatrics*.

[10] Bada H. S., Das A., Bauer C. R. (2002). Gestational cocaine exposure and intrauterine growth: maternal lifestyle study. *Obstetrics and Gynecology*.

[11] Szeto H. H. (1995). Opioid receptor approaches for the development of medications for pregnant women. *NIDA Research Monographs*.

[12] Stover M. W., Davis J. M. (2015). Opioids in pregnancy and neonatal abstinence syndrome. *Seminars in Perinatology*.

[13] Behnke M., Smith V. C., COMMITTEE ON SUBSTANCE ABUSE (2013). Prenatal substance abuse: short- and long-term effects on the exposed fetus. *Pediatrics*.

[14] Oji-Mmuo C. N., Corr T. E., Doheny K. K. (2017). Addictive disorders in women: The impact of maternal substance use on the fetus and newborn. *Neoreviews*.

[15] ACOG Committee on Health Care for Underserved Women (2012). Committee Opinion No. 524. *Obstetrics & Gynecology*.

[16] Weinberger S. M., Kandall S. R., Doberczak T. M., Thornton J. C., Bernstein J. (1986). Early weight-change patterns in neonatal abstinence. *American Journal of Diseases of Children*.

[17] Patrick S. W., Davis M. M., Lehmann C. U., Cooper W. O. (2015). Increasing incidence and geographic distribution of neonatal abstinence syndrome: United States 2009 to 2012. *Journal of Perinatology*.

[18] McGlothen-Bell K., Cleveland L., Recto P., Brownell E., McGrath J. (2020). Feeding behaviors in infants with prenatal opioid exposure: an integrative review. *Advances in Neonatal Care*.

[19] Dryden C., Young D., Campbell N., Mactier H. (2012). Postnatal weight loss in substitute methadone-exposed infants: implications for the management of breast feeding. *Archives of Disease in Childhood. Fetal and Neonatal Edition*.

[20] Shephard R., Greenough A., Johnson K., Gerada C. (2002). Hyperphagia, weight gain and neonatal drug withdrawal. *Acta Paediatrica*.

[21] World Health Organization (2006). *WHO Child Growth Standards: Length/Height-for-Age, Weight-for-Age, Weight-for-Length, Weight-for-Height, and Body Mass Index-for Age: Methods and Development*.

[22] Corr T. E., Schaefer E. W., Paul I. M. (2018). Growth during the first year in infants affected by neonatal abstinence syndrome. *BMC Pediatrics*.

[23] Favara M. T., Smith J., Friedman D. (2022). Growth failure in infants with neonatal abstinence syndrome in the neonatal intensive care unit. *Journal of Perinatology*.

[24] Liu A. J. W., Jones M. P., Murray H., Cook C. M., Nanan R. (2010). Perinatal risk factors for the neonatal abstinence syndrome in infants born to women on methadone maintenance therapy. *Australian and New Zealand Journal of Obstetrics and Gynaecology*.

[25] Bogen D. L., Hanusa B. H., Baker R., Medoff-Cooper B., Cohlan B. (2018). Randomized clinical trial of standard- versus high-calorie formula for methadone-exposed infants: a feasibility study. *Hospital Pediatrics*.

[26] Martinez A., Kastner B., Taeusch H. W. (1999). Hyperphagia in neonates withdrawing from methadone. *Archives of Disease in Childhood. Fetal and Neonatal Edition*.

[27] Charles M. K., Cooper W. O., Jansson L. M., Dudley J., Slaughter J. C., Patrick S. W. (2017). Male sex associated with increased risk of neonatal abstinence syndrome. *Hospital Pediatrics*.

[28] Unger A., Jagsch R., Baewert A. (2011). Are male neonates more vulnerable to neonatal abstinence syndrome than female neonates?. *Gender Medicine*.

[29] Holmes A. V., Atwood E. C., Whalen B. (2016). Rooming-in to treat neonatal abstinence syndrome: improved family centered care at lower cost. *Pediatrics*.

[30] Wachman E. M., Hayes M. J., Shrestha H. (2018). Epigenetic variation in OPRM1 gene in opioid-exposed mother-infant dyads. *Genes, Brain, and Behavior*.

[31] Kubisch K. A. Nutritional intake and weight gain in infants with neonatal abstinence syndrome: a literature review. https://stars.library.ucf.edu/honorstheses/561.

[32] Jansson L. M., Choo R., Velez M. L. (2008). Methadone maintenance and breastfeeding in the neonatal period. *Pediatrics*.

[33] Jansson L. M., Choo R., Velez M. L., Lowe R., Huestis M. A. (2008). Methadone maintenance and long-term lactation. *Breastfeeding Medicine*.

[34] Grossman M., Berkwitt A. (2019). Neonatal abstinence syndrome. *Seminars in Perinatology*.

[35] Weller A. E., Crist R. C., Reiner B. C., Doyle G. A., Berrettini W. H. (2021). Neonatal opioid withdrawal syndrome (NOWS): a transgenerational echo of the opioid crisis. *Cold Spring Harbor Perspectives in Medicine*.

