# Supplementary material for: Inpatient Growth in Infants Requiring Pharmacologic Treatment for Neonatal Opioid Withdrawal Syndrome
Source: Int J Pediatr. 2024 Aug 24;2024:2212688. doi: 10.1155/2024/2212688 (PMC11366048; doi:10.1155/2024/2212688)
Supplement: Supporting Information — Additional supporting information can be found online in the Supporting Information section. Table S1 Feeding intake by day of life and sex in infants with NOWS. [file 2212688.f1.docx]

**Feeding Intake by Day of Life and Sex (Supplementary Table S1)**

| **Day of life** | **Female**  **(mL/kg/day)** | **Male**  **(mL/kg/day)** | **p-value** | **Female**  **Kcal/kg/day** | **Male**  **Kcal/kg/day** | **P-value** |
| --- | --- | --- | --- | --- | --- | --- |
| **2** | 53.7 (5.19) | 60.5 (4.92) | 0.34 | 32.2(3.51) | 35.5 (3.32) | 0.50 |
| **3** | 86.3 (5.08) | 92.7 (4.89) | 0.36 | 58.9(3.43) | 59.2(3.30) | 0.96 |
| **7** | 169.6 (5.04) | 168.6 (4.79) | 0.89 | 112.3(3.41) | 112.7(3.23) | 0.94 |
| **11** | 187.0 (5.16) | 175.8 (4.85) | 0.12 | 126.7(3.50) | 119.2 (3.28) | 0.12 |
| **14** | 192.8 (5.24) | 183.4 (5.10) | 0.20 | 131.6(3.55) | 125.2 (3.46) | 0.19 |
| **18** | 201.5 (5.50) | 191.9 (5.60) | 0.22 | 135.0(3.73) | 130.6(3.80) | 0.41 |
| **21** | 200.0 (5.95) | 194.3 (6.20) | 0.50 | 133.5(4.04) | 133.3(4.22) | 0.97 |
| **25** | 201.3 (6.49) | 199.5 (6.36) | 0.84 | 135.1(4.41) | 134.6 (4.33) | 0.94 |
| **28** | 197.0 (7.26) | 191.5 (6.64) | 0.58 | 131.0(5.00) | 131.6(4.53) | 0.90 |
| **31** | 181.2(7.72) | 189.7 (7.09 | 0.42 | 119.2(5.27) | 127.8 (4.84) | 0.23 |
| **34** | 185.3 (8.51) | 194.4 (7.85) | 0.43 | 127.0 (5.82) | 129.5(5.36) | 0.76 |
| **37** | 181.1 (8.76) | 187.4(8.24) | 0.60 | 122.4 (6.00) | 126.8(5.63) | 0.59 |
| **41** | 166.1 (9.34) | 190.2 (8.72) | 0.06 | 112.5 (6.39) | 129.6 (5.96) | 0.05 |
| **44** | 155.4 (11.05) | 171.6 (9.29) | 0.26 | 105.4 (7.57) | 117.3(6.36) | 0.23 |
| **47** | 159.4 (11.05) | 168.6 (11.0) | 0.55 | 109.0 (7.57) | 120.1 (7.54) | 0.30 |
| **51** | 145.8 (13.34) | 154.9 (11.62) | 0.61 | 92.0 (9.15) | 109.9 (7.96) | 0.14 |
| **54** | 156.0 (13.34) | 151.6 (12.4) | 0.81 | 100.2 (9.15) | 103.4 (8.48) | 0.80 |
| **57** | 153.2 (18.57) | 157.1 (14.5) | 0.87 | 107.7 (12.7) | 106.6 99.96) | 0.95 |
| **61** | NA | 128.5 (18.53) | NA | NA | 87.4 (12.74) | NA |
| **64** | NA | 141.3 (22.58) | NA | NA | 95.1 (15.53) | NA |
